# Reports of acute adverse events in mRNA COVID-19 vaccine recipients after the first and second doses in Japan

**DOI:** 10.1038/s41598-022-19936-5

**Published:** 2022-09-15

**Authors:** Tetsuya Akaishi, Tamotsu Onodera, Tatsuya Takahashi, Hideo Harigae, Tadashi Ishii

**Affiliations:** 1 Department of Education and Supports for Regional Medicine, Tohoku University Hospital, Sendai, Japan; 2 Health and Welfare Department, Miyagi Prefectural Government, Sendai, Japan; 3 Department of Hematology, Tohoku University Graduate School of Medicine, Tohoku University, Sendai, Japan

**Keywords:** Medical research, Epidemiology

## Abstract

Mass vaccination against coronavirus disease 2019 (COVID-19) is ongoing in many countries worldwide. This study reports the occurrence of acute adverse events among vaccine recipients at a mass vaccination center in Japan. Between August and November 2021, approximately 130,000 individuals received two mRNA vaccine doses (mRNA-1273; Moderna) at the vaccination center. Acute adverse events at the site were observed in 1.1% of the recipients after the first dose and in 0.4% of the recipients after the second dose. The most common event was vasovagal syncope/presyncope, followed by acute allergic reactions. The occurrence rate of vasovagal syncope/presyncope was highest in the young population of those aged 16–29 years, but such age-dependency was not apparent in acute allergic reactions. Both symptoms were more prevalent in women than in men. Vasovagal syncope/presyncope occurred mainly within 20 min of the injection, whereas nearly half of the episodes of acute allergic reactions occurred after 20 min. The vaccine being injected while the recipient was in the supine position effectively reduced the occurrence of vasovagal syncope/presyncope. In summary, the suggested risk factors for vasovagal syncope/presyncope included a young age and female sex. The vaccine being injected while the recipient was in the supine position would reduce the risk of vasovagal syncope/presyncope.

## Introduction

Coronavirus disease 2019 (COVID-19), which is caused by severe acute respiratory syndrome coronavirus 2 (SARS-CoV-2), was the world’s largest public health concern in 2021^1^. To suppress the transmission of the infection, each country in the world has promoted a mass vaccination campaign for its nations since 2020^2,3^. Japan has also started vaccination campaigns for priority groups, including health workers, since February 2021, and Japan also started mass vaccination campaigns for all citizens in May 2021^4^. The effectiveness of the two doses of vaccines against the SARS-CoV-2 infection has been widely reported worldwide, with the estimated effectiveness of at least 50% in preventing infection before the Omicron (B.1.1.529) variant surge^5–8^. In addition to vaccine effectiveness against infection and hospitalization, safety and vaccine side effects have been also reported in many previous studies^9–11^. However, the acute adverse events of vaccination at the sites of mass vaccination centers remain largely unknown. This study reports the observed acute adverse events at the vaccination site in approximately 130,000 recipients who underwent vaccination at a local governmental mass-vaccination center, one of the largest in Japan, with the aim of further promoting safe and efficient mass vaccination projects at mass vaccination centers.

## Methods

### Participants and evaluated variables

**Figure 1:**
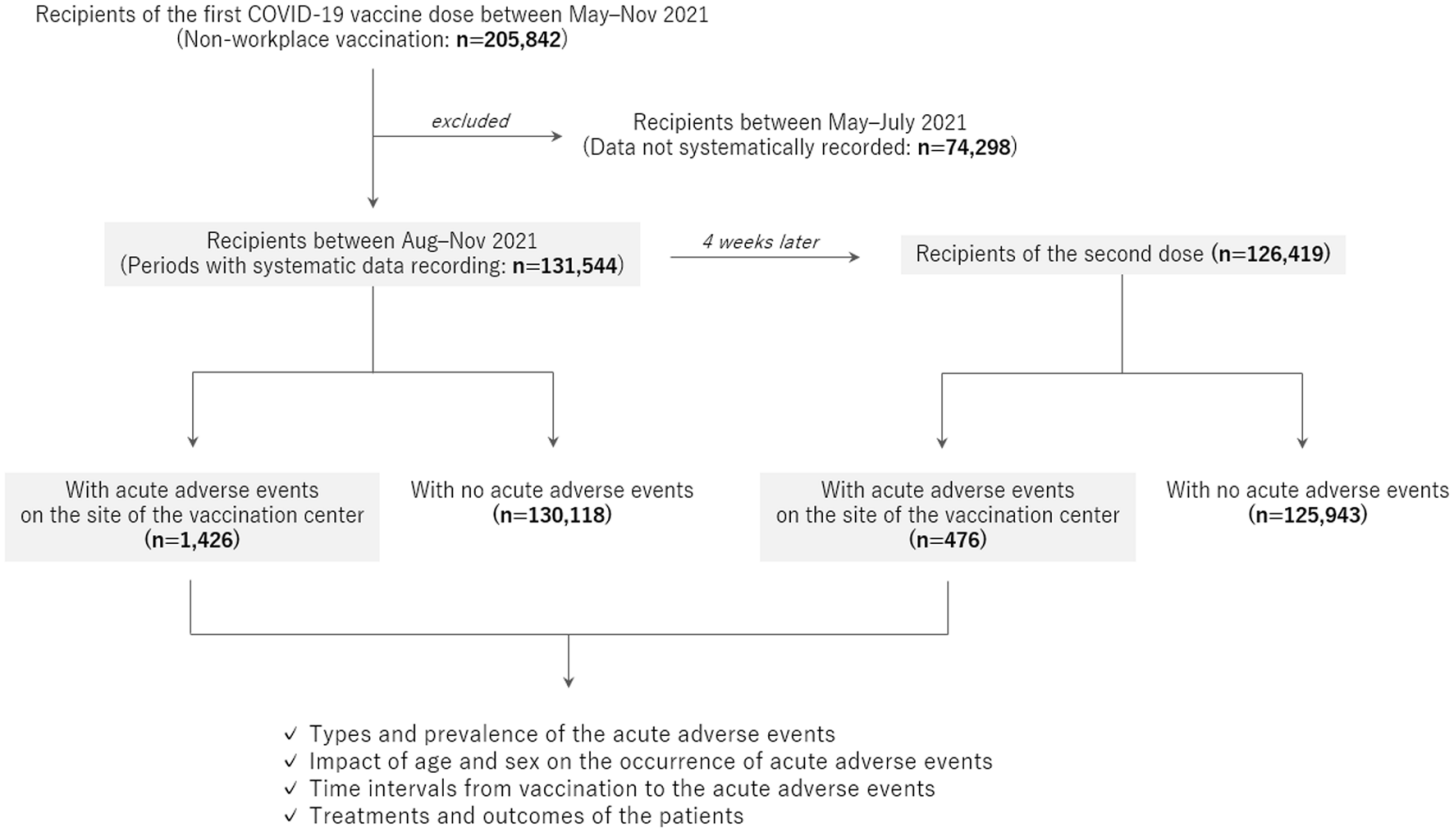
Flow diagram of the study design. All recipients of the Moderna mRNA vaccine against COVID-19 at a single mass vaccination center were enrolled in the study. Detailed information on the occurrence of acute adverse events at the site of the center was evaluated after stratification by age group, sex, and the time of vaccination.

The enrolled participants were recipients of the first and second doses of mRNA COVID-19 vaccines, mRNA-1273 (Moderna Corp, Cambridge, USA)^12^, who were vaccinated at a single large-scale mass-vaccination center located in Sendai City between August and November 2021. All the vaccine recipients were aged ≥ 16 years. Data on age and sex were collected from all recipients. Additional information regarding the details of the adverse events was collected from those who had acute adverse events at the vaccination center. The acute adverse events were categorized into the following diagnoses at the site by the doctors who examined the patients: vasovagal syncope/presyncope as a representative manifestation of immunization stress-related response, acute allergic reaction, anaphylaxis, and other conditions. The study design is illustrated in Fig. 1.

For reference, individuals with a previous medical history of vasovagal syncope/presyncope, including those with that after the first COVID-19 vaccine dose, were recommended to receive the vaccine dose in the supine position, laying on the bed in the first-aid office of the testing center. Therefore, the recipients who had vasovagal syncope/presyncope after the first vaccine dose received the second vaccine dose in the supine position. Information on the type of body position used during the vaccine injection (supine or normal sitting position) was collected.

### Observation time on site after injection

All of the vaccine recipients were asked to remain in the follow-up booth of the vaccination center for at least 15 min after the injection, and those individuals who had past medical histories of severe food or drug allergic reactions, including anaphylaxis, were asked to stay in the follow-up booth for at least 30 min after the injection. Individuals who were anxious about the development of acute adverse events were also advised to stay at the follow-up booth for at least 30 min. After the first vaccine dose, all recipients were then guided to another booth to be explained the next vaccine dose schedule and to make a reservation for the second vaccine dose. Therefore, almost all vaccine recipients after the first vaccine dose stayed at the vaccination center for at least 30 min. Meanwhile, many recipients of the second vaccine dose could have left the center before 30 min after the injection.

### Statistical analysis

Qualitative variables were compared between the two groups using the chi-square test or Fisher’s exact test, according to the sample size of each cell. The 95% confidence interval (CI) was further calculated for the prevalence of each symptom among all vaccine recipients. As multiple comparisons were performed simultaneously, p < 0.001 was considered statistically significant in this study. Statistical analyses were performed using the R statistical software (version 4.0.5; R Foundation for Statistical Computing, Vienna, Austria).

### Ethical standards statement

This study was approved by the institutional review board of the Tohoku University Graduate School of Medicine (approval number: 2021-1-566). All processes of the present study were performed in accordance with the ethical standards of the Declaration of Helsinki of 1964 and its later amendments. Informed consent was obtained from all of the participants.

## Results

### Overall occurrence of acute adverse events on site

A total of 131,544 individuals aged ≥ 16 years were vaccinated with the first dose of the mRNA COVID-19 vaccine at the vaccination center during the study period. Among them, 126,419 (96.1%) were vaccinated for the second dose at the same center 4 weeks after the first dose. The demographics of the enrolled recipients and observed acute adverse events at the vaccination site are summarized in Table 1. Vasovagal syncope/presyncope was the most common acute event for both doses, followed by acute allergic reactions. The occurrence rate of vasovagal syncope/presyncope was much higher after the first dose than after the second dose (0.72% vs. 0.16%, p < 0.0001), but the rate of acute allergic reactions did not differ between the doses (0.07% vs. 0.06%, p = 0.2404). The occurrence rate of vasovagal syncope/presyncope did not significantly differ between daytime (9:30 a.m. to 5:00 p.m.) and nighttime (5:00 p.m. to 9:00 p.m.) for both the first dose (0.73% vs. 0.71%, p = 0.6250) and second dose (0.17% vs. 0.12%, p = 0.0630). A total of 2952 recipients underwent the vaccine injection in the supine position. The occurrence rate of vasovagal syncope/presyncope among those vaccinated in the supine position was lower than that among others vaccinated in the usual sitting position (0.07% vs. 0.46%, p = 0.0003).

**Table 1:** Demographics and observed acute adverse events at the vaccination site.

	After the first vaccine dose	After the second vaccine dose	P values
Number, n	131,544	126,419	–
Male, %	54.0%	53.9%	0.5824
16–49 years old, %	77.7%	77.4%	0.0649
50–64 years old, %	21.5%	21.8%	0.0988
65 + years old, %	0.8%	0.8%	0.3123
**Acute adverse events on the site of the vaccination center, n (%)**			
Total	1426 (1.08%)	476 (0.38%)	< 0.0001
Nausea	355 (0.27%)	92 (0.07%)	< 0.0001
Dizziness, vertigo	774 (0.59%)	172 (0.14%)	< 0.0001
Tinnitus, hearing problems	69 (0.05%)	9 (0.007%)	< 0.0001
Pharyngeal discomfort	47 (0.04%)	34 (0.03%)	0.2055
Palpitation	210 (0.16%)	79 (0.06%)	< 0.0001
Headaches	46 (0.03%)	27 (0.02%)	0.0399
Paresthesia in limbs	179 (0.59%)	64 (0.05%)	< 0.0001
Weakness of limbs	30 (0.02%)	7 (0.006%)	0.0002
Dyspnea/chest pain	89 (0.07%)	56 (0.04%)	0.0123
Abdominal pain	8 (0.006%)	7 (0.006%)	1.0000
Problems of the injection site	12 (0.009%)	7 (0.006%)	0.3611
**Diagnosis for the acute adverse events, n (%)**			
Vasovagal syncope/presyncope	952 (0.72%)	197 (0.16%)	< 0.0001
Acute allergic reaction	97 (0.07%)	78 (0.06%)	0.2404
Anaphylaxis	6 (0.005%)	5 (0.004%)	1.0000
**Treatment for the acute adverse events, n (%)**			
Oral medications	75 (0.06%)	64 (0.05%)	0.4845
Drip transfusion	13 (0.01%)	7 (0.006%)	0.2651
Intravenous steroid	2 (0.002%)	3 (0.002%)	0.6815
Intramuscular adrenaline injection	0 (0.0%)	1 (0.001%)	0.4901
Emergency transfer to the hospital	5 (0.004%)	3 (0.002%)	0.7270
Emergency transfer with anaphylaxis	0 (0.0%)	1 (0.001%)	0.4901

The demographic data of the whole recipients of the mRNA COVID-19 vaccines and clinical manifestation and treatments in those with acute adverse events seen on the site of the vaccination center are compared between the first and second vaccine doses. The percentage for each symptom or diagnosis is for the number of overall individuals who received the first or second dose.

### Age of individuals with adverse events on site

**Figure 2:**
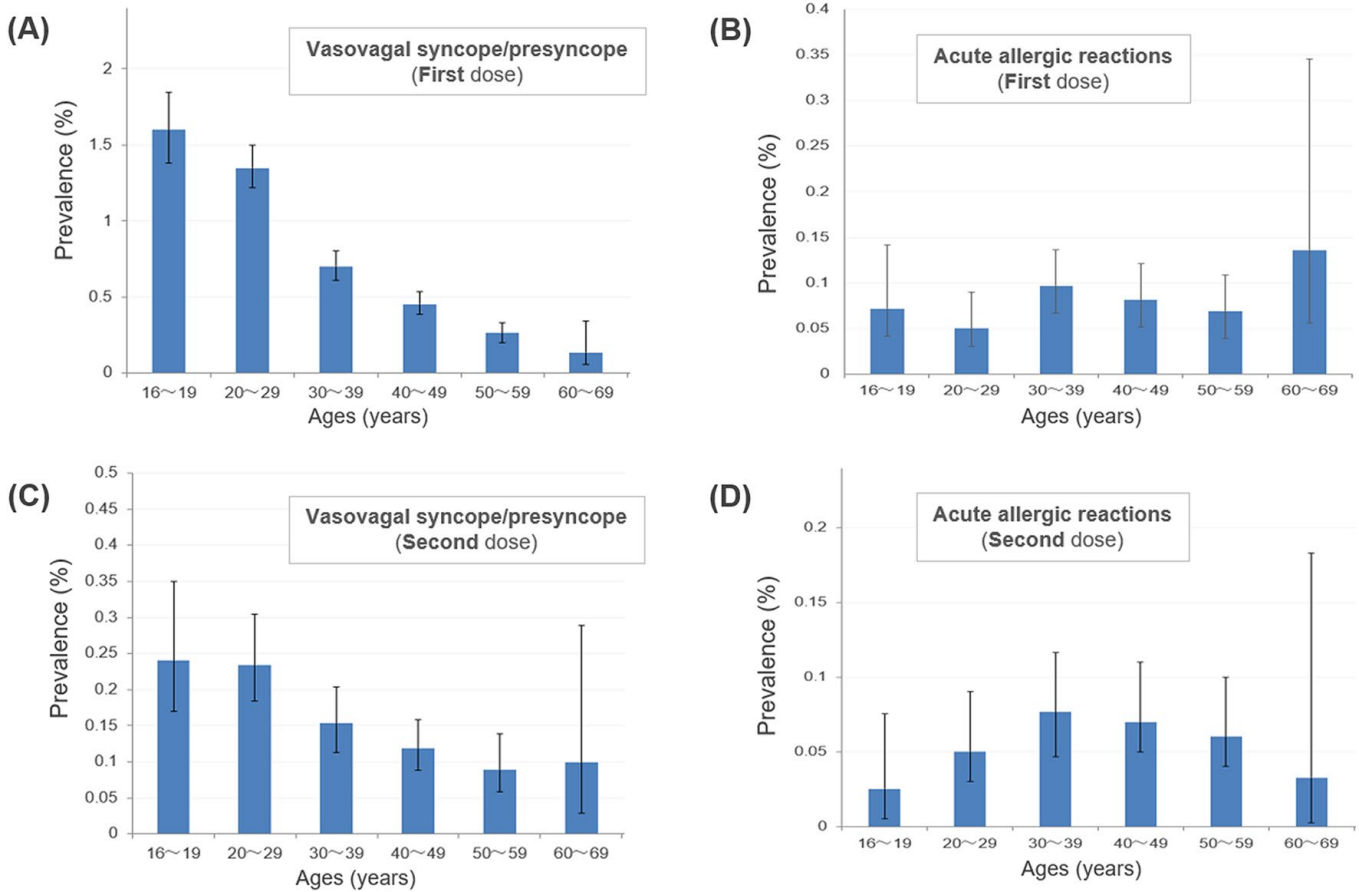
Occurrence rate of acute adverse events by age group. The prevalence and 95% confidence interval of acute adverse events (vasovagal syncope/presyncope or acute allergic reaction) after the first vaccine dose (**A**, **B**) and after the second vaccine dose (**C**, **D**) in different age groups are shown. The occurrence of vasovagal syncope/presyncope was most likely to occur in younger populations of those aged 16–29 years, whereas the occurrence of acute allergic reactions did not demonstrate such a remarkable age dependency. The figure was created using Microsoft Office Excel 2016 software (https://www.microsoft.com).

The prevalence of vasovagal syncope/presyncope and acute allergic reactions by age group is shown in Fig. 2. The occurrence rate of vasovagal syncope/presyncope was significantly highest in the younger population aged 16–29 years, particularly after the first vaccine dose. Meanwhile, the occurrence of acute allergic reactions did not show a remarkable age dependency. When comparing between men and women, the rates of vasovagal syncope/presyncope were slightly higher in women for both the first vaccine dose (male vs. female: 0.60% vs. 0.88%, p < 0.0001) and the second vaccine dose (0.09% vs. 0.23%, p < 0.0001). The occurrence rates of acute allergic reactions were much higher in females for both the first dose (0.02% vs. 0.13%, p < 0.0001) and the second dose (0.03% vs. 0.10%, p < 0.0001).

### Timing of adverse events on site after injection

**Figure 3:**
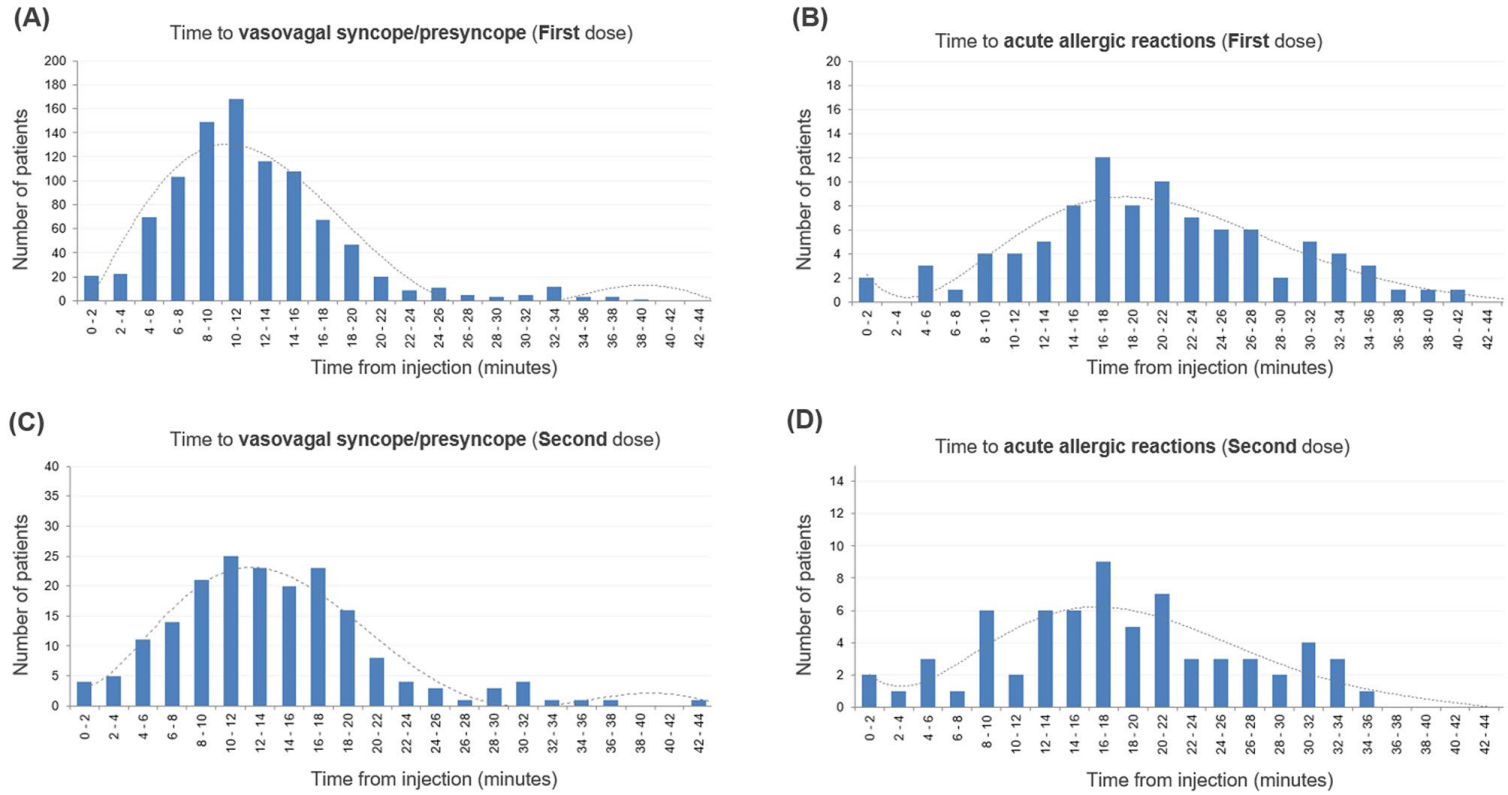
Timing of acute adverse event by age group. Histograms for the time from the vaccine injection to the occurrence of the acute adverse events after the first vaccine dose (**A**, **B**) and after the second vaccine dose (**C**, **D**) are shown. The peak time of occurrence of vasovagal syncope/presyncope was between 10 and 12 min after the injection, and that of acute allergic reactions was between 16 and 18 min after the injection. The figure was created using Microsoft Office Excel 2016 software (https://www.microsoft.com).

Distributions of the time interval from vaccination to the manifestation of acute adverse events are shown in Fig. 3. The peak for the occurrence of vasovagal syncope/presyncope occurred between 10 and 12 min after injection for both the first and second vaccine doses, whereas the peak for the occurrence of acute allergic reaction was between 16 and 18 min. To be noted, because some patients with acute allergic reactions may have left the vaccination center before 30 min after the injection, especially after the second vaccine dose, the peak for the occurrence of acute allergic reaction could be even later than 16–18 min after the injection.

### Occurrence of anaphylaxis

A total of 11 individuals (five after the first vaccine dose and six after the second dose) of the 257,963 vaccine recipients (0.004%) were clinically suspected to have anaphylaxis. Two of them were men, and nine of them were women. The elapsed time from receiving the vaccine ranged from 3 to 30 min, and the age ranged from 30 to 58 years. Although these 11 patients were clinically suspected to have anaphylaxis, treatment with adrenaline was required in only one individual (0.0004%). The individual who required adrenaline treatment was a 46-year-old woman who started to experience difficulty in breathing and swallowing 13 min after receiving the second vaccine dose, and she was thus transported to a nearby general hospital by ambulance.

## Discussion

The results of this study demonstrate that acute adverse events occurring at the vaccination center were reported in 1.1% of first-dose recipients and 0.4% of second-dose recipients. For both doses, almost all occurrences of vasovagal syncope/presyncope took place within 30 min of vaccination. The decreased occurrence rate of overall acute adverse events after the second dose compared to that after the first dose was mostly resulted from the decreased rate of vasovagal syncope/presyncope after the second dose. This fact implies that the risk of immunization stress-related responses was significantly higher after the first vaccine dose compared to that after the second vaccine dose. Furthermore, the present study demonstrated that the occurrence of vasovagal syncope/presyncope could be effectively avoided by performing the supine position injection. Vasovagal syncope/presyncope can be caused by stimulation of the parasympathetic nervous system and is a common adverse stress response to invasive or stressful events^13,14^. The suggested risks for the occurrence of vasovagal syncope/presyncope in this study, other than anxiety at the first dose, included a younger age (typically 16–29 years) and female sex. This was compatible with the previously reported characteristics for the epidemiology of vasovagal response^15^. A notable finding that may be helpful for future mass vaccination projects is that the occurrence of vasovagal response could be decreased by performing the injection in the supine position on the bed. The occurrence of vasovagal syncope/presyncope consumes time, manpower, and medical resources and may sometimes cause injuries to the recipients by falling down to the floor. Active utilization of the supine position injection should be considered a reliable and cost-effective measure for recipients who are strongly anxious about the injection. As the vasovagal response is known to recur in some individuals, especially in females, vaccine recipients with a past medical history of vasovagal response may also be candidates for an injection in the supine position^16^.

Another finding of this study was that the occurrence rate of acute allergic reactions did not differ after the first and second doses of vaccination. In addition, the occurrence of acute allergic reactions did not show age dependency, whereas it was significantly higher in women than in men. Women have been known to suffer from higher rates of adverse drug responses, including allergic reactions, than men^17,18^, which has been mainly explained from the aspect of pharmacokinetics or pharmacodynamics. The present study demonstrated that intramuscular injection of an mRNA vaccine also causes higher rates of acute allergic reactions in women than in men. This fact may suggest that females are predisposed to dysregulated immune response upon the first-time administration of drugs, as suggested by the higher prevalence of autoimmunity in women than in men^19^. To be noted, the reported rate of acute allergic reaction was significantly lower than that of approximately 2% as previously reported in the United States^20^. This discrepancy between the countries implies that the rate of acute allergic reactions with mRNA vaccines may differ with countries and ethnicities.

A limitation of this study was that it did not evaluate adverse responses after the recipients went home. Thus, the rate of allergic reactions, regardless of the time since vaccination, might have been much higher. Another limitation of this study was that past medical histories of recipients who had acute adverse events at the vaccination site could not be comprehensively collected. As a result, we could not statistically evaluate the impact of the past medical history on the occurrence of acute adverse events among the participants.

In conclusion, acute adverse events after receiving intramuscular mRNA vaccines against COVID-19 at the site of the mass vaccination center were reported in approximately 1% of the recipients, which was higher after the first dose than the second dose. Most of the events comprised vasovagal syncope/presyncope, which is possibly based on anxiety. The prevalence of vasovagal syncope/presyncope was higher in female and younger recipients than in male and older recipients. The prevalence of acute allergic reactions was also higher in women than in men, but it did not show an apparent age dependency. A supine position injection was useful for avoiding the occurrence of vasovagal syncope/presyncope, which should be actively utilized in recipients with a history of vasovagal syncope/presyncope or strong anxiety.

## Supplementary Information

Supplementary Information.

## Data Availability

The datasets used and/or analyzed during the current study are included in this published article and its supplementary information file (Supplementary Table 1).
